# Investigation of Acoustic Properties of Different Types of Low-Noise Road Surfacers under In Situ and Laboratory Conditions

**DOI:** 10.3390/ma15020480

**Published:** 2022-01-09

**Authors:** Janusz Bohatkiewicz, Maciej Hałucha, Marcin Kamil Dębiński, Michał Jukowski, Zbigniew Tabor

**Affiliations:** 1 Faculty of Civil Engineering, Cracow University of Technology, 31-155 Cracow, Poland; 2 Faculty of Civil Engineering and Architecture, Lublin University of Technology, 20-618 Lublin, Poland; m.halucha@pollub.pl (M.H.); m.debinski@pollub.pl (M.K.D.); m.jukowski@pollub.pl (M.J.); 3 Voivodeship Roads Authority in Katowice, 40-609 Katowice, Poland; zbigniew.tabor@kongresdrogowy.pl

**Keywords:** quiet surfaces, porous surfaces, sound absorption, tire/road noise, impedance tube, SPB

## Abstract

Current literature on the performance characteristics of road surfaces is primarily focused on evenness, roughness and technical durability. However, other important surface properties require analysis, including noisiness, which is an important feature of the environmental impact of vehicular traffic around roads. This can be studied using various methods by which road noise phenomena are investigated. The method used to measure the noise performance of road surfaces herein is the Statistical Pass-By (SPB) method, as described in ISO 11819-1:1997. The impedance tube method was used for sound absorption testing, as described in ISO 13472-2:2010. These tests were performed under a variety of conditions: in situ and in laboratory. The existence of relationships between them can be helpful in selecting surfaces for noise reduction. Preliminary surface noise tests can be performed in the laboratory with samples consisting of various compounds. This is less expensive and faster than doing so on purpose-built surfaces. The paper presents study results for sound absorption coefficients of various types of low-noise surfaces in in situ conditions (on an experimental section and on operated road sections) and in the laboratory setting. The results of the tests performed on the operational sections were compared to the results of the surface impact on road noise using the SPB method. The correlations between the test results help confirm the feasibility of road surface pre-testing in the laboratory and the relation to tests performed using the SPB method under typical operating conditions.

## 1. Introduction

Road noise is one of the most frustrating environmental phenomena for people. It also has a significant effect on the health of people living in the immediate vicinity of roads [1]. It can be reduced in various ways: in the sound emission zone (e.g., road surfaces), in the propagation zone (e.g., noise barriers) and at the recipient level (e.g., acoustic insulation of windows) [2,3]. However, limiting the noise generated in the sound emission zone by using quiet surfaces is often the best solution, allowing for measurable results [4,5,6]. However, it requires knowledge of the phenomena occurring at the interface between the vehicle wheels and the road surface as well as how to study them. Numerous publications have described these mechanisms and their associated parameters [7,8]. The most important of these are road surface texture [9] and sound absorption coefficient [10,11]. Both of these are related to what is known as tire/road noise.

Tire/road noise is generated by two groups of phenomena: mechanical phenomena and aerodynamic phenomena. The first are related to the vibrations of the tire and its components (radial and tangential vibrations of the tire tread elements, adhesive interactions, stick–slip phenomenon, tire sidewall vibrations, belting vibrations, etc.); the second are related to the dynamics of the gas medium in the contact area of the tire with the road (including sucking in and pumping out air through the tire tread, the formation of air resonance in the set of channels created by the tire grooves and uneven road surface, the horn effect, Helmholtz resonance and the air turbulence resulting from tire movement) [8,12].

One effective measure to reduce this type of noise, which can be used as one of the first tasks in noise protection, is the use of so-called quiet road surfaces. These primarily reduce tire/road noise, although they also reduce noise emissions from other elemental sources to some extent. These are alternative actions to noise barriers, which are still popular in many European countries [13].

The use of this type of surface makes it possible to reduce the emission of traffic noise, especially the tire/road noise that dominates at higher speeds [14,15,16,17]. The use of quiet surfaces on roads with higher traffic speeds therefore results in a greater reduction in acoustic impact than when vehicle speeds are lower (less than 50 km/h). Nevertheless, at lower speeds one should also expect a mitigating effect on communication noise’s impact on the environment. However, it would be lower than at high speeds, and in some cases, it more likely involves a change in frequency characteristics (towards lower frequency sound) than a reduction in the sound levels measured in the entire analyzed range. The scale of possible noise reduction after the application of quiet surfaces is further described, e.g., in [18].

There are two types of quiet surfaces based on their structure. These are impervious (closed) and porous (open) surfaces. They reduce road noise using various phenomena. In the case of sealed (non-porous) surfaces, the incident sound wave is almost entirely reflected. Only a small portion of the acoustic energy is absorbed, unlike porous surfaces. Noise reduction occurs in this case by shaping the texture (surface) accordingly, e.g., by using smaller-diameter aggregate. In the case of porous surfaces, some of the acoustic energy is converted into heat within the structure. Porous surfaces are able to both absorb, to some extent, the sound generated by an automobile engine and reduce the noise generated by the contact between vehicle wheels and the road surface. It should be added, however, that sound absorption is provided by the so-called open pores that are connected to the road surface. Those air spaces that are not internally connected to each other (being closed) do not cause sound absorption [5,19]. For the impedance tube test, the angle of incidence of the wave is 90 degrees. When analyzing with other methods, the possible angle of incidence of the sound wave must be considered when testing and estimating the sound absorption coefficient. There are literature studies that confirm the effect of the incidence angle on the value of the sound absorption coefficient [20].

Road noise is affected by the texture of the road surface. Correct modeling of asphalt mix composition during the design stage affects the surface texture. By recognizing the relationship between the proportions of the components in the asphalt mixture and the properties of the texture, it is possible to determine the acoustic properties of surfaces at the design stage [21]. The use of surfaces to reduce the acoustic impact, especially new and untested solutions, is expensive and very time-consuming. It generally requires the construction of a test (pilot) section. Based on the results of these tests, it becomes possible to determine the surface features and the level of noise reduction produced. This article attempts to determine the correlation between the results of tests performed in laboratory conditions, on test sections and under road operating conditions with typical vehicular traffic. For this purpose, sound absorption coefficient studies were performed using impedance tubes, and the effect of the surface on road noise using the SPB method. The analytical results can help reduce road surface testing time by performing some testing under laboratory conditions using samples taken from the surface or made for that purpose. At the same time, they can be helpful in the acoustic diagnosis of operational surfaces that lose their acoustic durability over time.

Noisiness tests are performed in order to determine the acoustic properties of road pavements and their applicability for road noise protection. The Statistical Pass-By (SPB) score, which determines the road surface noisiness, is a popular method. Testing by this method to compare results requires equal conditions to be provided or appropriate correction factors to be introduced to bring conditions to a reference level [8]. This method has been used in many research projects, such as SILENCE WP F4. It concerns road surfaces, but takes into account a number of other factors responsible for noise generation that may affect the results. The test method for the sound absorption coefficient is different. In this case, the sound absorption coefficient is tested directly. This method concerns only a single parameter, and the test results are not affected by other external factors. Combining these two approaches provides a complete picture of the acoustic characteristics of road surfaces. The derivation and determination of exact relations between the results of these methods will allow for the design of surfaces by including the acoustic parameters, as well as the subsequent monitoring of the surface condition along with the acoustic parameters. Deterioration of the acoustic properties of road surfaces may be an indication of the need for maintenance, renovation or complete replacement of the road structure. This paper presents the results of tests with both methods and a comparison of these results with the derivation of preliminary relationships.

## 2. Methodology for Testing the Absorption Coefficient of Road Surfaces Using an Impedance Tube

**Figure 1:**
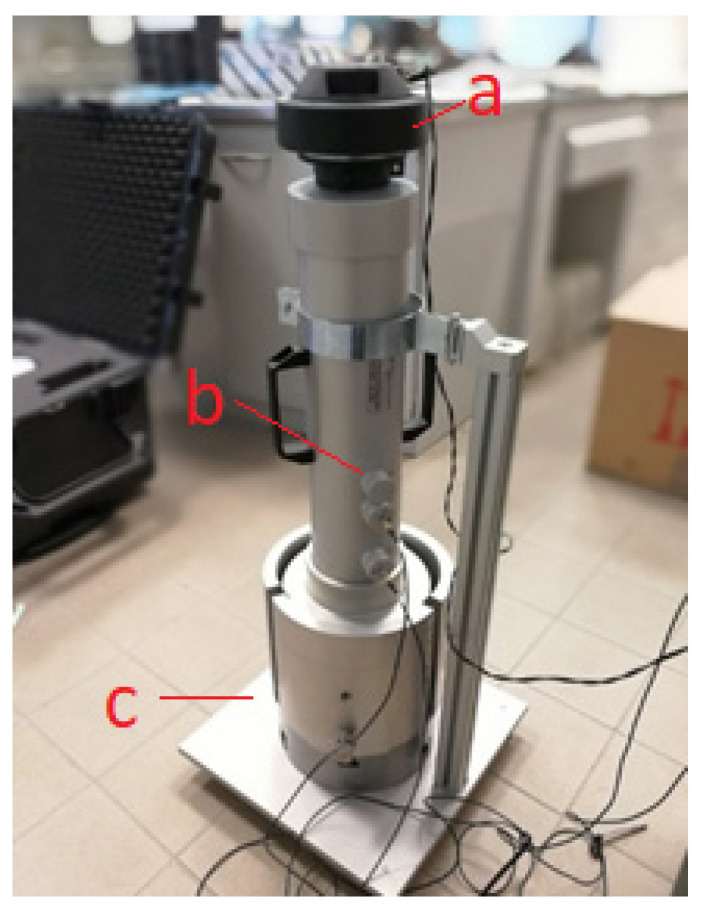
View of test stand used for testing the sound absorption coefficient of road surfaces in laboratory conditions. (**a**) Loudspeaker; (**b**) Microphones; (**c**) Sample tested.

Measurements of the sound absorption coefficient of road surfaces were made with an impedance tube, using the procedure described in ISO 13472-2 [22]. This method is non-destructive and does not require sampling of the road surface. It is intended primarily for smooth and reflective surfaces, for which the sound absorption coefficient at each center frequency of the 1/3 octave bands is no greater than 0.15. The results are presented in 1/3 octave frequencies ranging from 250 to 1600 Hz [22]. The results obtained by the foregoing method can be compared with the results obtained by laboratory testing using surface samples in the form of excised cores, according to the procedures described in documents [23,24,25]. However, they cannot be directly compared with the results of tests performed in a reverberation chamber using the method described in the standard [26], because in the method using an impedance tube, we are dealing with a planar wave incident perpendicularly on the tested surface, while the sound is dispersed in the “reverberation” method. Detailed requirements for measuring instruments are described in standards [22,24]. This equipment (AVEC, Blacksburg, VA, USA) consists of a signal generator, a sound source, an impedance tube, two microphones mounted flush with the inner wall of the tube in well-defined positions, an additional in situ device to hermetically connect the tube to the test surface, and a signal processing unit to perform the Fourier transformation on two channels simultaneously. A test stand designed at the Road and Bridge Research Institute was used to perform the tests. In addition to the equipment used for the in situ measurements, the test setup consisted of an additional bracket that allowed the impedance tube to be mounted on the test specimens (Figure 1).

The laboratory stand was constructed within the framework of the research project RID—I/76 Protection Against Road Noise, carried out under the Development of Road Innovations program by the consortium of the Cracow University of Technology, Warsaw University of Technology, Wrocław University of Technology, Road and Bridge Research Institute and Lublin University of Technology, commissioned by the General Directorate for National Roads and Motorways (GDDKiA) and the National Centre for Research and Development (NCBiR).

## 3. Methodology for Studying the Effect of the Surface on Road Noise Using the SPB Method

The procedure for testing the effect of the surface on road noise using the statistical pass-by method (SPB) is described in ISO 11819-1 [27]. It is intended to evaluate the vehicle noise generated on various road surfaces under specified traffic conditions. Measurements, using this procedure, are made for a large number of vehicles traveling on the road under real-world conditions. The results obtained are normalized to standard speeds by category or type of road tested. In this method, it is important to properly select the test vehicles so that they meet the requirements specified in the standard. It should also be noted that the results are affected by various external factors, such as road surface temperature and vehicle park [28]. Accordingly, detailed annexes to the test procedure have been developed in various countries to adequately account for these variables in the results [29].

**Figure 2:**
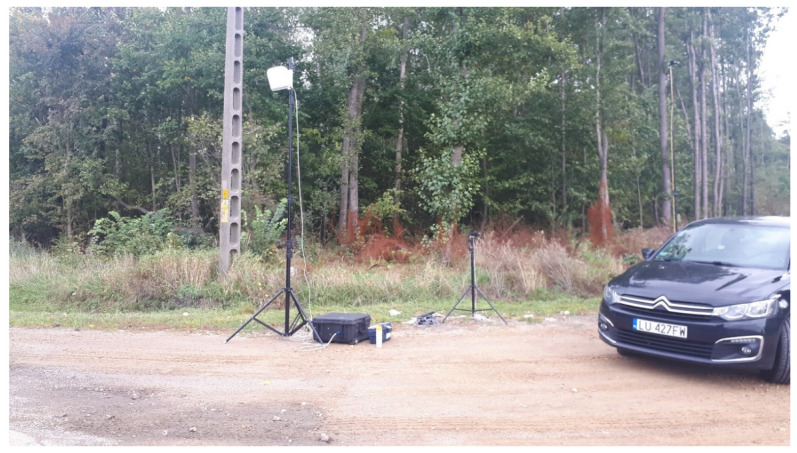
View of the test stand used for the SPB testing method in in situ conditions.

The test is performed at a distance of 7.5 m from the axis of the lane under analysis, and the microphone is placed on a tripod at a height of 1.2 m from the lane. Figure 2 shows the location of a sample measurement point.

By using this method, the noisiness of the surface can be determined, and the surface can be classified into the appropriate group in terms of its loudness [30]. By studying the results of tests in subsequent years of operation, it is also possible to assess the acoustic durability of road surfaces (maintenance of acoustic properties over time) and to take possible maintenance measures [31].

The result of a road pavement noise test performed using the SPB method is the Statistical Pass-By Index (SPBI). The results of tests performed using this method are presented later in the paper, as the difference between SPBI indices for road sections equipped with noise-reducing surfaces and sections equipped with surfaces without such properties (reference surfaces).

The advantages and disadvantages of both methods are apparent when comparing them. The impedance tube method tests the material properties of the road surface without taking other parameters into account, whereas several parameters are taken into account in the SPB method. In SPB, it is important to set up the measuring equipment so that the results are not affected by the geometric parameters of the road. However, in the case of sound absorption coefficient tests, roadside measurements are made directly on the roadway or from samples taken from boreholes, which requires stopping traffic on a given lane and providing appropriate protection. The SPB method requires obtaining an adequate sample size so that it is statistically significant. This is difficult to verify during testing, and therefore testing must be done with planned margins. In the case of the tube method, the test can be applied only within a limited range of the sound absorption coefficient. Both methods have advantages, limitations and disadvantages. Using these together provides a better picture of the acoustic performance of road surfaces.

## 4. Characteristics of the Tested Road Surfaces

The study was performed in two stages. The first stage included measurements with an impedance tube in Bolimów and on samples taken from the surface in situ. In the second stage, the study was carried out in the Silesian Voivodeship on four sections. SPB measurements and an in situ sound absorption coefficient test were performed. Table 1 presents a summary of test sections, with information on the tests performed.

Measurements of the sound absorption coefficient of road surfaces were made on more than a dozen selected road sections. They were selected in such a way that the tested surfaces were characterized by various acoustic properties (mainly in terms of absorption of the sound wave falling on them). First, measurements were taken on a test stretch in Bolimów near Warsaw (Poland), performed under the “Innovative Technology for Road Surfaces with Reduced Noise Emission” project as carried out by a consortium of Warsaw University of Technology, Mostostal Warszawa S.A. and Road and Bridge Research Institute and co-financed by the National Centre for Research and Development.

As a part of the aforementioned task, test sections were designed and constructed with surfaces that exhibited reduced noise levels. These sections were made of the following surface types [32]:

- Section No. 1—surface made of mineral and asphalt mixture AC 11
- Section No. 2—OGFC 11 (open-graded friction courses—a mixture with open structure, used in the USA)
- Section No. 3—surface made of porous asphalt PA 11
- Section No. 4—OGFC 8 (open-graded friction courses—a mixture with open structure, used in the USA)
- Section No. 5—surface made of SMA 8 mastic-grits mix
- Section No. 6—surface made of porous asphalt PA 8
- Section no. 7—surface made of SMA 5 mastic-grits mix

They consisted of a 4 cm thick wearable layer (various types of mixtures), a sealing layer, a 6 cm thick binder layer of AC 16 W asphalt concrete, and a mechanically stabilized substructure (the existing surface is about 20 cm thick). It should be noted that although these surfaces were characterized by reduced noise, not all of them had enhanced sound absorption properties. Some of them (e.g., SMA 5 and SMA 8) had other features affecting noise reduction (the macrotexture in these cases). A higher sound absorption coefficient was found for the surfaces that were porous, with plenty of voids (OGFC and PA). The reference surface, which in this case was an AC 11 mineral and asphalt mix, had a void content of 3.2%.

**Figure 3:**
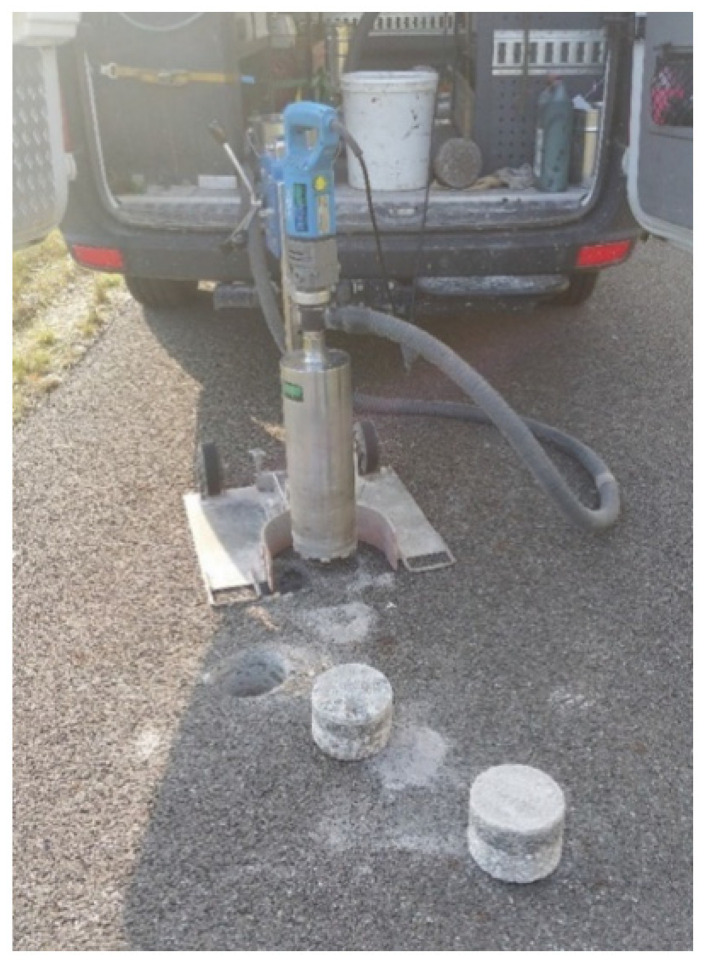
View of samples taken at the site of measurements in the research section in Bolimów.

**Figure 4:**
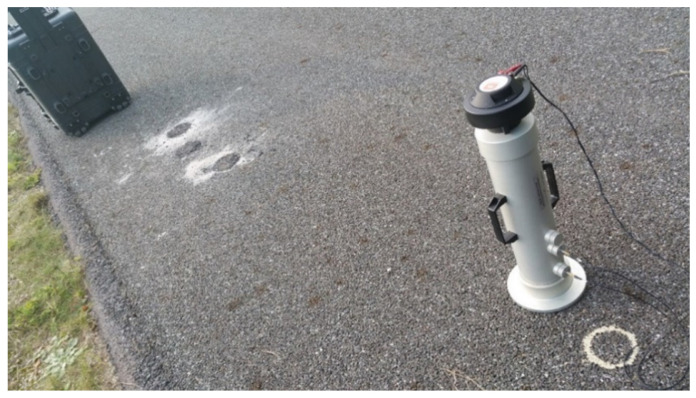
View of the location of the impedance tube during testing in relation to the surface sample collection site.

Three samples were collected from each test section (Figure 3) and analyzed under laboratory conditions. The samples had a diameter of 15 cm, which allowed a smaller diameter impedance tube of 10 cm to be placed on them. These were taken in close proximity to the site of the in situ test (Figure 4), so that correlations between the field and laboratory results could be studied.

Samples taken from the surface were then tested under laboratory conditions. Three samples were tested for each surface. The purpose of these measurements was to compare the results obtained under in situ and laboratory conditions for the same surfaces and to confirm the equivalence of these methods.

Tests were also performed on operational road sections with typical vehicular traffic. Four sections located in the Silesian Voivodeship in southern Poland were selected:

- Section A—voivodeship road No. 790 in the town of Niegowonice
- Section B—voivodeship road No. 793 on the Janów—Złoty Potok section
- Section C—voivodeship road No. 793 on the Żarki—Myszków section
- Section D—voivodeship road No. 790 in the town of Biskupice

The wearable layer of these road sections was made of BBTM 8S thin layer asphalt concrete, highly modified with polymers (an SBS elastomer—styrene-butadiene-styrene copolymer—was used in the asphalt production process). The measurements made on these road sections were intended to compare the results of the sound absorption coefficient tests with the results of the surface noise impact tests performed using the SPB method.

## 5. Results of Studies on the Absorption Coefficient of Road Surfaces and the Effect of Surface on Road Noise for Selected Road Sections

**Figure 5:**
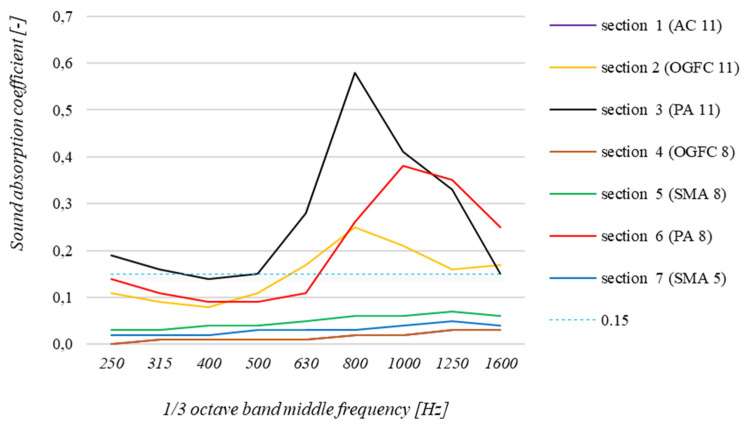
Summary of results of in situ sound absorption coefficient measurements for surfaces applied in the test section in Bolimów.

First, sound absorption coefficient tests were performed under in situ conditions for the seven road surfaces used on the test section in Bolimów. The results of these tests are shown in Figure 5, with the results for successive 1/3 octave band middle frequencies shown in each case after averaging performed in accordance with the requirements of the standard [24]. In the graph, the dashed line shows the sound absorption coefficient value of 0.15, which defines the precision limit of the measurement method.

By analyzing the test results shown in the Figure 5, one should conclude that PA 11, PA 8 and OGFC 11 surfaces have the best car noise absorption properties. The sound absorption coefficients measured under in situ conditions at each analyzed frequency reach high values. The common feature of these three surfaces is the high content of free spaces in their structure, which directly relates to the obtained peak values of the sound absorption coefficient in the 630–1250 Hz range. OGFC 8 is a surface that has a slightly lower proportion of voids. However, the results of the sound absorption coefficient measurement are surprisingly low (these results were later confirmed in the laboratory by repeating the measurement on collected samples, which is described in more detail later in this article). This phenomenon may be caused by contaminants that entered and clogged the pores in its structure. This is a common cause of the decrease in noise-reducing properties of porous surfaces [33]. Such surfaces require specialized cleaning procedures that involve removing contaminants using water under high pressure. The presence of bitumen on the non-wearing aggregate grains due to the test section being closed to traffic may also have caused this situation [34].

The other surfaces used on the test section in Bolimów (SMA 5 and SMA 8) do not have enhanced sound absorption properties compared to the reference AC 11 surface. However, this does not prove that they do not have sound-reducing properties. They are characterized by a smaller maximum dimension of the aggregate used compared to the reference surface. Consequently, the sound reduction properties achieved after their application are caused by other mechanisms related to their macrotexture (as further discussed in Section 1). Noise reduction is achieved in these cases by avoiding large irregularities (the so-called peaks) in the surface macrotexture. Nevertheless, it is important that such surfaces are not too even and smooth, as this can lead to a reduction in airflow at the point of contact with the vehicle wheels, which in turn leads to amplification of the so-called horn effect and consequently to an increase in tire/road noise. Having considered the foregoing, both of these effects must be taken into account when designing road surfaces. It should also be mentioned that tire/road noise increases on level and smooth surfaces when there is skidding motion (e.g., the stick–slip phenomenon) [8,35].

**Figure 6:**
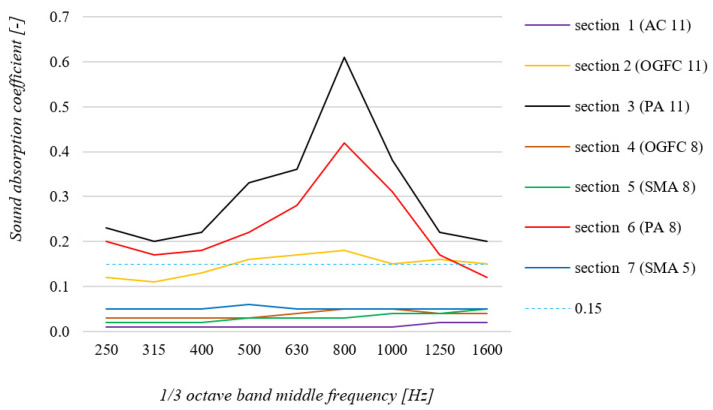
Summary of sound absorption coefficient measurements results, as taken under laboratory conditions using samples taken from the test section in Bolimów.

Tests under laboratory conditions were then performed for the same surfaces. Samples taken from each section on the same day as the in situ tests were used. In each case, tests were performed for three samples; Figure 6 shows the results averaged at each 1/3 octave frequency according to the procedure described in the standard [24]. The selection of laboratory sample diameters was based on a literature review. The diameter of the specimens was determined so as to avoid the interference and inaccuracies associated with an inadequate dimensions [36].

When analyzing the results of tests performed under laboratory conditions on samples collected from the research section in Bolimów, similar conclusions can be drawn as in the case of tests performed under in situ conditions. The three porous surfaces (PA 11, PA 8 and OGFC 11) have the best sound absorption properties. The other surface samples tested (SMA 5, SMA 8) have a much lower sound absorption coefficient, similar to the AC 11 reference surface. The conclusion regarding the lower-than-expected sound absorption coefficient values of the OGFC 8 surface, as mentioned above, can also be confirmed.

The tests under laboratory conditions were also designed to check the correlation of the results obtained under in situ conditions and the tests performed in the laboratory. It can be concluded that the results of the tests performed under field conditions are consistent with the results of the laboratory tests. In most cases, the difference between the absorption coefficient measurements at each frequency is small and far less than the 0.1 value. Larger differences can be observed for porous surfaces characterized by high sound absorption coefficients: PA 11 (maximum difference of 0.18), PA 8 (maximum difference of 0.18) and OGFC 11 (maximum difference of 0.07). These differences are due to the specifics of the measurement method [24], which is less precise when testing surfaces for which the sound absorption coefficient is greater than 0.15 (dashed line in Figure 5 and Figure 6). For the other surfaces, these differences were much smaller and were 0.03 for OGFC 8, SMA 8 and SMA 5 surfaces and 0.01 for the AC 11 surface.

The coefficient of determination R^2^ was calculated for all tested road surfaces, taking into account the correlations of the results obtained at each middle frequency of the 1/3 octave bands. The results of these calculations are shown below in Table 2.

When analyzing the data presented in the table above, it can be concluded that for each tested frequency (from 250 to 1250 Hz), there is a very high correlation between the results of the tests performed in in situ conditions and in the laboratory conditions. The exception is 1600 Hz, for which this correlation is lower. Nevertheless, it is important to recognize that the results of the tests performed in situ and in the laboratory conditions are very well correlated. The convergence of the results is satisfactory, and thus it can be argued that the location of the study does not affect the results obtained. It should be noted, however, that this article did not analyze samples specifically produced under artificial conditions but those taken from actual surfaces. On the other hand, their production method (appropriate compaction, texture representation) may affect the correlation of the results of tests performed in in situ conditions and in laboratory conditions. It is a direction in which further research should be conducted.

**Figure 7:**
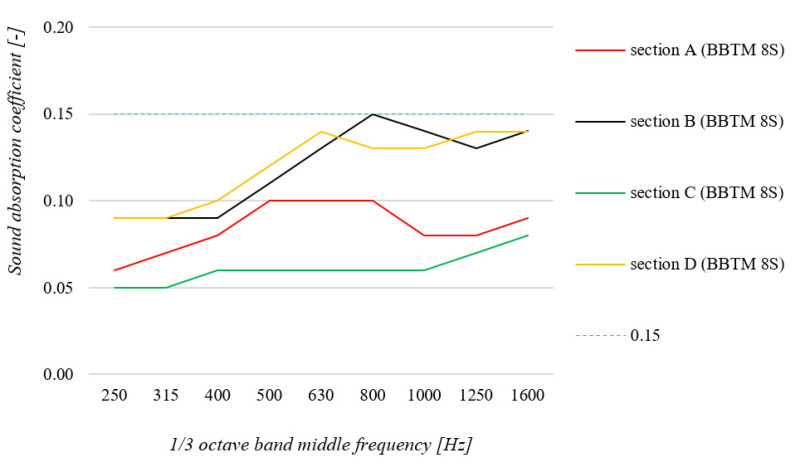
Summary of sound absorption coefficient measurements results in in situ conditions for surfaces in use in the Silesian Voivodeship.

This was followed by in situ testing on several selected road sections carrying typical vehicular traffic. The tests were performed for four road sections with a BBTM 8S type surface highly modified with polymers (detailed description is presented in Section 4). The measurement results are shown in Figure 7.

By analyzing the results of the in situ tests performed on the operational surfaces, it can be concluded that they were characterized by better sound absorption properties in relation to the closed surfaces used on the test section in Bolimów and in relation to the OGFC 8 surface. Sound absorption coefficients of about 0.05 to about 0.15 can be considered good, given that these are operational surfaces exposed to significant pollution from vehicular traffic. In contrast, the results obtained for these road sections are lower than PA 8, PA 11 and OGFC 11 surfaces, which are highly porous and thus cumbersome to apply in real-world conditions.

**Figure 8:**
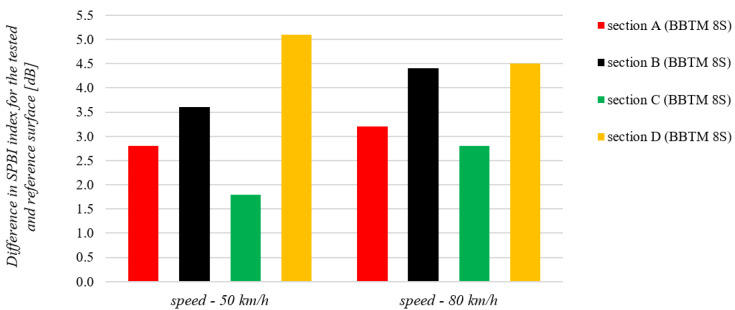
Summary of sound absorption coefficient measurements results in in situ conditions for surfaces in use in the Silesian Voivodeship.

A study of the effect of the surface on road noise using the SPB method was performed for the same sections of voivodeship roads, currently operated in a typical manner. In each case, tests were performed in the vicinity of sections equipped with a BBTM 8S surface and in the vicinity of adjacent sections equipped with reference surfaces that do not have road noise reduction properties. These were SMA 11 mineral and asphalt surfaces. The results of these tests are shown in Figure 8.

The results obtained with regard to the surface effect on road noise (SPB method) confirmed the conclusions drawn from the sound absorption coefficient results. Each tested surface had good road noise reduction properties. They range from 1.8 dB to 5.1 dB at 50 km/h and from 2.8 dB to 4.5 dB at 80 km/h. It should also be emphasized that the results of the SPB method reflect the influence of all phenomena occurring during vehicular traffic (described in detail in chapter 1), not only the sound absorption by road surfaces. The high correlations between the results of the sound absorption coefficient measurements using the impedance tube and the results of the tests performed using the SPB method demonstrate the strong effect of surface type on road noise emissions. Correlation values are presented in Table 3, taking into account the division into vehicle speeds for which the SPB tests were performed and the center frequencies of the 1/3 octave bands.

There are high correlations between the results of the sound absorption coefficient and surface effects on road noise. The R^2^ determination coefficients reach slightly higher values for 50 km/h (from 0.63 to 0.96) compared to 80 km/h (from 0.53 to 0.86). Nevertheless, it should be noted that for both vehicle speeds that are considered in the SPB method, there are strong correlations between the results of these tests.

## 6. Discussion

Based on the sound absorption coefficient results obtained, a correlation analysis was performed between the results obtained in situ (α_s_) and in the laboratory (α_L_). The correlation study showed a strong correlation between the results of surfaces tested in situ and samples collected from cores tested in the laboratory. Based on the results of the R correlation test, it was decided to derive a linear relationship between the test results for each band. A linear model of the general form shown in the formula below was adopted.

α_s_ = a × α_L_ + b (1)

where:

α_s_—sound absorption coefficient measured in-situ (-)

α_L_—sound absorption coefficient measured in the laboratory (-)

a,b—coefficients of the equation defined in Table 4 according to the given frequency (-)

This function converts the results obtained during laboratory testing to the values that would be obtained during in situ testing on a given surface. The model included the laboratory result in a given band (α_L_), as well as a coefficient a and a constant b. The function parameters were obtained using multiple regression techniques. The results for each band are shown in Table 4.

Based on the results, it can be concluded that the best correlation and coverage of the results based on R^2^ was achieved for the 1000 Hz band (R = 0.987 and R^2^ = 0.974). This indicates very highly correlated in situ and laboratory test results. The lowest result was obtained for the 1600 Hz band (R = 0.762 and R^2^ = 0.581). This result no longer shows a correlation as strong as the other bands, but it can be considered statistically significant. Comparing the value of the standard error of estimate, the smallest value was obtained for the 315 Hz band, 0.01506. The largest error was recorded for the 800 Hz, with 0.06904. From the results obtained, it can be concluded that the highest correlation was obtained for the bands 250 Hz, 315 Hz, 400 Hz and 500 Hz, at low values of standard error of estimate. The other bands retained high correlation scores, but the standard error of the estimate is much larger. All results can be considered statistically significant and valid. Normal distribution analysis of the residuals was performed to confirm the significance of the obtained models. In all cases, a *p* > 0.05 value was obtained, and the hypothesis of normal distribution of the residuals was confirmed.

The foregoing tests indicate strong relationships between sound absorption coefficient test methods in situ and in laboratory conditions. Accordingly, the relationships between the difference in SPBI indices for the reference surface and the test surface and the average sound absorption coefficient were derived for the tested road surfaces. Linear regression analysis was used to do this. These relationships are shown below:

ΔSPBI (50 km/h) = 35.5·*α* (dB) (2)

ΔSPBI (80 km/h) = 38.5·*α* (dB) (3)

where

ΔSPBI—difference of the SPBI index between the reference surface and the tested surface calculated on the basis of the test results according to the standard [27] from relation (1) for reference speed 50 km/h or 80 km/h (dB).

A—average sound absorption coefficient of the road surface in the frequency range from 250 to 1600 Hz (-).

The R^2^ determination coefficient for these relationships has very high values: 0.97 for 50 km/h and 0.99 for 80 km/h. In contrast, the standard errors are 0.7 dB and 0.3 dB, respectively. Using Equations (2) and (3), it is therefore possible to calculate the noise reduction expressed by SPBI from the results of the sound absorption coefficient tests, which can be performed both on existing surfaces and under laboratory conditions on their samples. However, it should be noted that the tested road surfaces, for which these relationships were derived, were characterized by high porosity (they had free spaces in their structure). Sound absorption is therefore one of the most important parameters for these surfaces to determine their noise performance. Other phenomena occurring at the wheel–surface interface that generate tire/road noise have less of an effect on reducing noise emitted to the road vicinity for this type of surface.

It should also be noted that this study was performed for only one type of surface wearable layer (BBTM 8S highly modified with polymers). The results should not be directly related to other surfaces for which test results and their correlations may differ. However, it is presumed that these differences will not be significant for porous surfaces. Similar tests should be performed for other porous surfaces used to reduce road noise.

## 7. Summary and Conclusions

This paper presents the results of sound absorption coefficient tests using an impedance tube made for several road surfaces on a test section in Bolimów. Samples were taken from these sections and studied under laboratory conditions using a test stand designed and constructed at the Road and Bridge Research Institute. These sections and test samples were selected in such a way that they were characterized by different acoustic properties (mainly in terms of absorption of the incident sound wave).

Analyzing the results of the study, it was found that three of the tested surfaces, PA 11, PA 8 and OGFC 11, have higher vehicle noise absorption properties. The high content of voids was a common feature of these surfaces, found in their structures. The surface that had a slightly lower proportion of voids than the above was OGFC 8. However, the sound absorption coefficient measurement results were surprisingly low in this case. This could have been caused by contaminants that entered and clogged the pores in its structure or by the presence of bitumen on the grains of non-wearing aggregate, due to the fact that the studied road section was closed to traffic. When analyzing the results of tests performed under laboratory conditions on the collected samples, similar conclusions were made as in the case of tests performed under in situ conditions. Three porous surfaces (PA 11, PA 8 and OGFC 11) had the best sound absorption properties. The sound absorption coefficients for the other tested surfaces were similar to the AC 11 reference surface.

The results of measurements under laboratory conditions were found to be highly consistent with the results of measurements made under field conditions. The difference between the results at each frequency was far less than the 0.10 value in most cases. Larger differences were observed for porous surfaces characterized by high sound absorption coefficients: PA 11 (maximum difference of 0.18), PA 8 (maximum difference of 0.18) and OGFC 11 (maximum difference of 0.07). These differences were due to the specific nature of the measurement method (ISO 13472-2, 2010), which has less precision for surface measurements for which the sound absorption coefficient is greater than 0.15. For the other surfaces, these differences were much smaller and were 0.03 for OGFC 8, SMA 8 and SMA 5 surfaces and 0.01 for the AC 11 surface. It was also found that there was a very high correlation between the results of tests performed in in situ conditions and in the laboratory. The R^2^ coefficients of determination reached values greater than 0.81 for every 1/3 octave band middle frequency tested (with 1600 Hz being the exception, for which the R^2^ coefficient was lower).

Sound absorption coefficient tests were also performed under in situ conditions on several selected road sections with typical vehicular traffic. The tests were performed for four road sections with a BBTM 8S–type surface, highly modified with polymers (a detailed description is presented in Section 4). For the same road sections, the surface effects on road noise were studied using the SPB method. There are high correlations between the results of these studies as well. The R^2^ determination coefficients reached slightly higher values for 50 km/h (from 0.63 to 0.96) compared to 80 km/h (from 0.53 to 0.86). Nevertheless, it was found that for both vehicle speeds considered in the SPB method, there were strong correlations between the results of the tests. Relationships were derived based on the ability to calculate the noise reduction expressed by the SPBI for these surfaces, following the results of sound absorption coefficient tests performed under in situ or laboratory conditions.

The results of the foregoing tests and their correlations indicate that it is possible to pre-test the noise performance of porous surfaces under laboratory conditions. These tests can be performed using the method described in ISO 10534-2 [24] on samples taken from these surfaces. It is important to note, however, that the method used to make the sample (proper compaction, texture shaping, etc.) can affect the results of these tests. In addition, it should be noted that the tested road surfaces were characterized by high porosity and were made of one type of mixture (in the case of surfaces operated in real conditions). The results presented in the paper and the correlations between them therefore apply only to porous surfaces. Closed surfaces experience different phenomena that result in noise reduction as compared to porous surfaces. Additionally, correlations between test results for surface types other than BBTM 8S (highly modified with polymers) may vary. Still, one should not expect a significant difference between them. However, it is advisable to expand the research sample to include other surface types, since they were not studied as part of this paper.

In conclusion,

- By analyzing the results of the study, it was found that three of the tested surfaces, PA 11, PA 8 and OGFC 11, have higher vehicle noise absorption properties. The high content of voids was a common feature of these surfaces, found in their structures.
- The results of measurements under laboratory conditions were found to be highly consistent with the results of measurements made under field conditions. The difference between the results at each frequency was far less than the 0.10 value in most cases.
- It was found that there is a very high correlation between the results of the tests performed in in situ and under laboratory conditions. The R^2^ coefficients of determination reached values higher than 0.81 for each tested center frequency of the 1/3 octave bands.
- There are high correlations between the SPB test results and the sound absorption coefficient test. The R^2^ determination coefficients reach slightly higher values for 50 km/h (from 0.63 to 0.96) compared to 80 km/h (from 0.53 to 0.86).
- The results of the study and their correlations with each other indicate that it is possible to pre-test the noisiness of porous surfaces under laboratory conditions. These tests can be performed using the method described in ISO 10534-2 [14] on samples taken from these surfaces.

## Figures and Tables

**Table 1:** Overview of testing plots.

Section	Surface Type	Sound Absorption Coefficient (In Situ)	Sound Absorption Coefficient (Laboratory)	SPB
1	AC 11	Yes	Yes	No
2	OGFC 11	Yes	Yes	No
3	PA 11	Yes	Yes	No
4	OGFC 8	Yes	Yes	No
5	SMA 8	Yes	Yes	No
6	PA 8	Yes	Yes	No
7	SMA 5	Yes	Yes	No
A	BBTM 8S	Yes	No	Yes
B	BBTM 8S	Yes	No	Yes
C	BBTM 8S	Yes	No	Yes
D	BBTM 8S	Yes	No	Yes

**Table 2:** Correlation of the road surface sound absorption test results, as performed under in situ and laboratory conditions.

**Frequency (Hz)**	250	315	400	500	630	800	1000	1250	1600
**Determination Coefficient R^2^**	0.95	0.95	0.91	0.90	0.81	0.91	0.97	0.86	0.58

**Table 3:** Correlation of the road surface sound absorption test results, as performed under in situ conditions (Figure 6), and the surface effect on road noise (SPB) (Figure 7).

Determination Coefficient R^2^	**Speed (km/h)**	**1/3 Octave Band Middle Frequency (Hz)**								
		250	315	400	500	630	800	1000	1250	1600
	50	0.86	0.82	0.96	0.85	0.86	0.64	0.64	0.77	0.63
	80	0.83	0.74	0.86	0.72	0.74	0.62	0.58	0.66	0.53

**Table 4:** Results of multivariate regression tests for individual bands.

Band (Hz)	Parameter of the Equation		R	R^2^	Standard Error of Estimate
	a	b			
250	0.823	−0.00759	0.976	0.953	0.01809
400	0.556	0.00491	0.956	0.913	0.01591
630	0.651	0.00686	0.899	0.807	0.04838
1000	1.153	−0.00015	0.987	0.974	0.03055
1600	0.978	0.01627	0.762	0.581	0.06125

## Data Availability

Not applicable.
